# Loss of Heterozygosity and Its Importance in Evolution

**DOI:** 10.1007/s00239-022-10088-8

**Published:** 2023-02-08

**Authors:** Caiti Smukowski Heil

**Affiliations:** 1 Department of Biological Sciences, North Carolina State University, Raleigh, NC, USA

**Keywords:** Loss of heterozygosity, Experimental evolution, *Saccharomyces*, Hybrids

## Abstract

Loss of heterozygosity (LOH) is a mitotic recombination event that converts heterozygous loci to homozygous loci. This mutation event is widespread in organisms that have asexual reproduction like budding yeasts, and is also an important and frequent mutation event in tumorigenesis. Mutation accumulation studies have demonstrated that LOH occurs at a rate higher than the point mutation rate, and can impact large portions of the genome. Laboratory evolution experiments of heterozygous yeasts have revealed that LOH often unmasks beneficial recessive alleles that can confer large fitness advantages. Here, I highlight advances in understanding dominance, fitness, and phenotypes in laboratory evolved heterozygous yeast strains. I discuss best practices for detecting LOH in intraspecific and interspecific evolved clones and populations. Utilizing heterozygous strain backgrounds in laboratory evolution experiments offers an opportunity to advance our understanding of this important mutation type in shaping adaptation and genome evolution in wild, domesticated, and clinical populations.

## Introduction

Experimental evolution aids evolutionary biologists in their fundamental goal of connecting genotype to phenotype to fitness. Towards this goal, the community has identified and linked changes in ploidy, copy number variants, aneuploidy, and single nucleotide variants to fitness in a number of different organisms, but particularly in microbial systems like *E. coli* and *S. cerevisiae* (Lenski 2017; McDonald 2019). While many experimental design setups of microbial systems are done in homozygous haploids or diploids, an increasing number of experiments have utilized heterozygous intraspecific (Ament-Velásquez et al. 2022; Burke et al. 2014; Phillips et al. 2020, 2022; Smukowski Heil et al. 2017, 2019; Wing et al. 2020) and/or interspecific hybrids (Bautista et al. 2021; Charron et al. 2019; Dunn et al. 2013; Peris et al. 2020; Piotrowski et al. 2012; Smukowski Heil et al. 2017, 2019; Vázquez-García et al. 2017). Heterozygous strain backgrounds introduce more complex genetic variation and interactions, and can better represent dynamics relevant in a number of natural and anthropogenic environments. The targets of selection often change, and a new class of mutations known as loss of heterozygosity (LOH) becomes detectable. LOH describes a mitotic mutational event in which a heterozygous locus or loci become(s) homozygous. While LOH has long been appreciated to occur, it is receiving increased attention due to an appreciation of how frequently LOH occurs, the proportion of the genome it impacts, and the large effect it has on phenotypes and fitness.

LOH is typically described based on the tract length of the homozygous portion of the genome. Interstitial LOH events, also known as gene conversions, result in short stretches of homozygosity (typically less than 10 kb). In contrast, terminal LOH events result from reciprocal crossovers or break-induced repair during mitotic cell division, often encompassing large physical stretches (often more than 100 kb) of the chromosome extending to the telomere. LOH is appreciated to occur frequently in organisms with asexual life cycles, including Daphnia, yeasts and other fungi, and clonally propagated crops (L.-Y. Chen et al. 2019; Ene et al. 2019; Flynn et al. 2017; James et al. 2009; Magwene et al. 2011; Peter et al. 2018; Schoustra et al. 2007). It is also a particularly important mutational event in cancers (Aguilera and Gómez-González 2008; Jeggo et al. 2016).

LOH underlies the two-hit model of tumorigenesis, which describes a mutation occurring in one allele of a tumor suppressor gene, followed by the loss of the wild type allele via LOH (Hartwell and Smith 1985; Knudson 1971; Lasko et al. 1991).

Mutation accumulation studies in *Saccharomyces cerevisiae* have demonstrated that the rate of LOH is incredibly high, occurring at a rate of 0.3–5.6 × 10^−2^ per cell division for interstitial LOH and 1.420139.3 × 10^−3^ per cell division for terminal LOH (Dutta et al. 2021; Sui et al. 2020). This translates to an average LOH rate varying between 2.6 and 7.1 × 10^−5^ per SNP per cell division, much higher than the rate of point mutations (1–3 × 10^−10^ per base pair per cell division for diploids) (Dutta et al. 2017, 2021; Sharp et al. 2018; Sui et al. 2020; Zhu et al. 2014). Distributions of interstitial LOH and terminal LOH differ from each other, and from meiotic associated gene conversions and crossovers. Terminal LOH events are enriched near telomeres, whereas interstitial LOH are fairly evenly distributed, suggesting differences in either the formation and/or the resolution of these events (Sui et al. 2020).

Rates of LOH depend on genetic background, level of heterozygosity, ploidy, and genomic region (Dutta et al. 2021, 2022; Pankajam et al. 2020; Sui et al. 2020; Tutaj et al. 2022). Particular regions of the genome are prone to LOH, especially the *S. cerevisiae* rDNA locus on chromosome XII, where SNPs located near the telomere have a LOH rate of 1.6 × 10^−4^ per cell division (Sui et al. 2020). The proportion of the genome impacted by LOH can vary dramatically; in one mutation accumulation experiment, an average of 15.9% of the genome experienced LOH, with some lines experiencing near genome-wide LOH (Dutta et al. 2021). The rate of LOH also increases with ploidy, with triploids having 2.2 × 10^−2^ events per cell division, and tetraploids having 8.4 × 10^−2^ events per cell division, with a skewed proportion of events being short and interstitial LOH compared to diploids (Dutta et al. 2022). While most of the mutation accumulation studies have focused on *S. cerevisiae*, rates of LOH have also been documented in mutation accumulation studies of other asexual species. In species of yeasts in the Saccharomycodaceae family, rates of LOH range from 2 to 11 × 10^−6^ per SNP per cell division (Nguyen et al. 2020). In *Daphnia pulex*, the rate of LOH is about 8 × 10^−8^ per SNP per generation, and interestingly most of the LOH events are deletions (Flynn et al. 2017).

Population genomic surveys and mutation accumulation studies have thus clearly demonstrated that LOH occurs frequently in the lab and in nature. To understand the phenotypic and fitness consequences of these events, we can turn to the growing body of literature from experimental evolution. Typically laboratory evolution experiments using heterozygous yeasts have maintained cultures asexually, but a number of experiments now incorporate a sexual cycle (Burke et al. 2014; Leu et al. 2020; McDonald et al. 2016; Phillips et al. 2020, 2022). For this review, I will focus on detecting LOH in asexual populations, however, it is important to note that LOH may shape the genome evolution of populations that have cycles of asexual and sexual reproduction by altering allele frequencies during the asexual growth phase, and through generation of LOH following return to growth (RTG) after abortive meiosis (Brion et al. 2017; Dayani et al. 2011; Laureau et al. 2016; Mozzachiodi et al. 2021). LOH is formed during this process by returning cells that had initiated meiosis to rich nutrients before the commitment to complete meiosis. Double-strand breaks formed during meiotic prophase I are unrepaired or repaired as crossovers or non-crossover gene conversions, and cells resume mitotic cell division without reducing ploidy, such that the cell starts with four potentially recombined chromosomes that then split between mother and daughter to maintain diploidy (Esposito and Esposito 1974; Sherman and Roman 1963; Simchen et al. 1972). This can generate extensive LOH in intraspecific hybrids, and even in sterile interspecific hybrids (Laureau et al. 2016; Mozzachiodi et al. 2021). This review will primarily focus on laboratory evolution and detection of LOH using *Saccharomyces* species, but many principles extend to other asexual organisms.

### Experimental Evolution Demonstrates LOH Can be Adaptive and Promote Emergence of Numerous Phenotypes

Asexual evolution of heterozygous yeasts have added several important insights. First, as identified in recent mutation accumulation studies, LOH is commonly detected in evolved clones and populations. One experiment found an average of 5.2 LOH events per clone after ~500 generations of evolution of *S. cerevisiae* populations (James et al. 2019). Other studies have found lower numbers of LOH events, but this may be due to an under detection of smaller LOH events, in addition to other factors like genetic background, number of replicates, and amount of heterozygosity in the ancestral genotype.

LOH events provide a particularly fascinating lens to understanding dominance of alleles in evolving populations. The probability of fixation of a mutation is dependent on its selection coefficient and dominance, and thus, mutations with higher dominance are more likely to establish, a concept known as Haldane’s sieve. However, in asexual diploids where LOH is common, recessive beneficial mutations can escape Haldane’s sieve by becoming homozygous via LOH. For example, Gerstein et al. showed that recessive mutations for *S. cerevisiae* nystatin drug resistance frequently experienced LOH when incubated in the presence of nystatin (Gerstein et al. 2014). These LOH events were advantageous and rose to high frequency in populations, exhibiting a pattern more typical of dominant mutations. Theoretical work demonstrates that LOH highly reduces the time to fixation for a recessive beneficial allele in asexual populations, and when rates of LOH are higher than the mutation rate, can equal or even outpace the rate of fixation in sexually evolving populations (Mandegar and Otto 2007). There is some empirical evidence for selection of advantageous LOH events for presumed recessive or partially recessive alleles (see below), although the dominance of the alleles under selection was not explicitly tested.

However, it’s apparent from several studies that dominance is also impacting experimentally evolved populations in more complex ways. A recent study identified that a key mutation in the gene *ACE2* in the route to the *S. cerevisiae* multicellularity phenotype known as “snowflake” yeast is underdominant. However, LOH of the underdominant genotype (*ACE2*/*ace2)* rapidly promotes the rise of the beneficial *ace2/ace* multicellular phenotype during laboratory evolution, facilitating the crossing of a fitness valley (Baselga-Cervera et al. 2022). Overdominant variants, in contrast, by definition suffer a fitness loss if heterozygosity is lost, and this can constrain evolutionary trajectories for linked partially dominant or recessive alleles. For example, when partially dominant mutations in *S. cerevisiae WHI2* are linked to overdominant mutations in *STE4*, adaptive LOH at *WHI2* is reduced, and when it does occur, promotes varied compensatory copy number and point mutations (Fisher et al. 2021). Mutations in the gene *CCW12* are another potential example of overdominance constraining LOH occurrence (Johnson et al., 2021; Leu et al. 2020). Fixed mutations in *CCW12* are enriched in evolved diploids and rarely experience LOH, despite being located in a LOH hotspot on the right arm of chrXII.

Recessive deleterious mutations impose more extreme constraints on LOH, perhaps facilitating the maintenance of heterozygosity in some circumstances. This is particularly well illustrated in a 10,000 generation evolution experiment of *S. cerevisiae* haploids and diploids (Johnson et al., 2021). While the founders of the populations were isogenic, many de novo mutations that arose and fixed as heterozygotes in diploids were predicted to be high impact mutations in essential genes, compared to this class of mutations being almost completely absent in fixed alleles in haploids and homozygous diploids. The accumulation of these likely recessive deleterious alleles seemingly prevented LOH from occurring in these regions, maintaining heterozygosity. Recessive lethal mutations do not appear to be segregating at high frequency in wild strains of *Saccharomyces*, in which spore viability is typically very high (Duan et al. 2018; Magwene et al. 2011), but recessive deleterious alleles are predicted to be accumulating in domesticated strains, many of which have lost the ability to complete meiosis (De Chiara et al. 2022). In *Candida albicans*, which is an obligate diploid, several recessive lethal alleles have been identified that limit specific LOH events (Feri et al. 2016). Wild isolates of *C. albicans* have notedly high heterozygosity, and this offers an interesting hypothesis to further examine (Bensasson et al. 2019).

Several studies have used genetic modifications to demonstrate that some of the LOH events detected in evolved populations are at high frequencies due to positive selection. For example, in *S. cerevisiae × S. uvarum* hybrids evolved in low phosphate, we observed species-specific LOH at the phosphate transporter *PHO84* dependent on temperature. Allele replacement experiments showed that loss of the *S. cerevisiae* allele results in a 39.30% fitness increase at 15 °C, whereas the loss of the *S. uvarum* allele at the same locus increases fitness by 25.57% at 30 °C (Smukowski Heil et al. 2017, 2019). A complementary study crossed the *S. cerevisiae* gene deletion collection to *S. uvarum* to create a collection of hemizygous hybrids and tested their fitness under several nutrient limiting conditions in pooled competitive fitness assays (Lancaster et al. 2019). This approach identified several beneficial *S. uvarum* hemizygous genes underlying two different LOH events in hybrids evolved in low phosphate (Smukowski Heil et al. 2017). More broadly, these experiments found that beneficial LOH events were typically specific to one environmental condition, which is consistent with a number of studies that have shown LOH events to be repeatable across replicates in the same environment, but rarely if ever across environments.

Using CRISPR-mediated LOH, James et al. found that a highly repeatable LOH event in a high salt environment encompassing the salt efflux pump *ENA* has a fitness advantage of 27% (James et al. 2019). In this particular example, the favored allele is a result of historic introgression in *S*. *cerevisiae* from *S. paradoxus*, providing an example of adaptive introgression. James et al. also targeted *MAL31*, one of the maltose metabolism genes that experienced LOH in beer wort evolved strains, but identified a non-significant increase in fitness (4%). While clear that some LOH events are associated with a large increase in fitness, more work is needed to disentangle how common these examples are. Though tedious, CRISPR-mediated LOH is a promising tool to be able to further examine fitness effects of other LOH events (Sadhu et al. 2016). CRISPR LOH could also help resolve if observed LOH result from beneficial alleles in one gene or multiple genes.

LOH also plays a significant role in domesticated yeast evolution. LOH is observed in isolates of *Saccharomyces cerevisiae* from a wide variety of ecological niches, but is most frequently observed in human-associated niches, which are more likely to be heterozygous (Duan et al. 2018; Magwene et al. 2011; Peter et al. 2018). Strains associated with beer, bread, and other fermentations often show a high degree of admixture (Peter et al. 2018). Humans may have promoted outcrossing in these strains by bringing diverse strains together, and/or there may be selection for admixture in these environments. LOH is often identified in ale and lager yeasts (Libkind et al. 2011; Monerawela et al. 2015; Nakao et al. 2009; Saada et al. 2022; Salazar et al. 2019). Laboratory evolution of *S. cerevisiae × S. eubayanus* allotetraploids, created to mimic the lager yeast *S. pastorianus*, demonstrated that LOH can produce industrially relevant phenotypes like acquired flocculation and loss of maltotriose utilization (Gorter de Vries et al. 2019). A unique experiment carried out by sequencing serially repitched tetraploid ale yeast at several North American breweries found that LOH occurs independently and repeatedly on chrVIII, chrXII, and chrXV (Large et al. 2020). The allele frequency change on chrVIII is estimated to yield a 5.7% increase in fitness. While mutation accumulation experiments suggest that LOH is increased in tetraploids (Dutta et al. 2022), evidence from evolution experiments is too limited to determine how this impacts tetraploid genome evolution and fitness. In the above examples, the LOH events identified were almost exclusively larger, terminal LOH, whereas in the mutation accumulation lines, LOH events in higher ploidies were biased toward smaller, interstitial LOH. Whether this is due to underdetection of smaller LOH events in these populations, fitness effects, or other confounding factors is unclear. Further studies incorporating higher ploidies are therefore needed, particularly as polyploidy is a hallmark of domesticated crops (as well as fermentations) and important in human tumorigenesis.

Finally, LOH appears to play an important role in fungal pathogen phenotypes including drug resistance (Beekman and Ene 2020; J. Chen et al. 2017; Coste et al. 2006; Cowen et al. 2000; Dunkel et al. 2008; Forche et al. 2005). In vivo and in vitro evolution of *C. albicans*, a prevalent opportunistic pathogen in the human gastrointestinal tract, identified frequent LOH and a highly increased mutation rate in regions adjacent to LOH due to mutagenic effects of recombination (Ene et al. 2018; Forche et al. 2009). Other in vitro studies observing in host evolution have identified recurrent LOH at genes including efflux pumps and drug targets (Ford et al. 2015). Similar associations between LOH and pathogenic phenotypes have been identified in other fungi, including *Cryptococcus* (Dong et al. 2019; Michelotti et al. 2022; Stone et al. 2019) and chytrid fungus *Batrachochytrium dendrobatidis* (James et al. 2009).

### Detecting LOH in Evolved Populations

Results from experimental evolution demonstrate that LOH is a frequent and important mutation event in heterozygous organisms. Detecting LOH in evolved populations is contingent on the amount of heterozygosity present in the ancestor. If a goal of an experiment is to examine LOH, parental strains should be chosen to maximize heterozygosity. If heterozygosity is low, results should take into account that some small LOH events are likely to go undetected. In Saccharomyces, studies attempting to identify mitotic and meiotic gene conversion events typically cross two haploid strains to generate a parent with 50,000 to 140,000 heterozygous SNPs (James et al. 2019; Liu et al. 2018, 2019).

The pipeline for detecting LOH in evolved clones or populations entails following a typical variant calling pipeline, then analyzing allele frequencies. Most studies utilize the best practices pipeline for variant calling outlined by GATK (McKenna et al. 2010). The first step in detecting LOH is to identify quality heterozygous sites in the ancestor that are expected to be heterozygous in evolved clones/populations. For many experiments, this will involve sequencing haploid parents, which allows phasing of alleles as well. Standard parameters include using GATK SelectVariants to focus the analysis on SNPs and exclude indels, and VariantFiltration parameters “QD < 2.0 || FS > 60.0 || MQ < 40.0 || MQRankSum < −12.5 || ReadPosRankSum < − 8.0” and a minimum read depth (DP) of 20. For a haploid, the genotype (GT) should be 0/0 (homozygous reference allele) or 1/1 (alternate reference allele), and for a heterozygous diploid, GT should be 0/1.

After variants are called in the evolved clones/populations, filter sites to specifically include only the ancestral heterozygous sites. This can be done with tools like BEDtools “intersect” (Quinlan and Hall 2010). Standard practice is to identify LOH tracts by identifying SNPs that deviate from the average allele frequency, then using consecutive SNPs with the same allele frequency to distinguish LOH blocks. A minimum of three consecutive SNPs is typically applied for detecting LOH. In a clone, this would be three consecutive SNPs with an allele frequency at or close to 0 or 1. Several studies have LOH scripts available (Marsit et al. 2021; Peter et al. 2018), and the programs Y_MAP_ and Control-FREEC can also identify and plot LOH (Abbey et al. 2014; Boeva et al. 2012).

There is still considerable difficulty to call LOH events in the case that there are more than two heterozygous haplotypes, which can frequently occur in some yeast strains such as the ale beer brewing yeasts. For these scenarios, utilizing long read sequencing technology and phasing tools like nPhase may be necessary (Abou Saada et al. 2021). A number of recent successful efforts have been made to phase *S. cerevisiae* genomes (Fay et al. 2019; O’Donnell et al. 2022; Saada et al. 2022).

### Special Considerations with Interspecific Hybrids

There are a number of considerations that must be taken into account when working with interspecific hybrids. First, while different species of *Saccharomyces* will readily mate with one another, only a small fraction results in viable F1s (Bendixsen et al. 2022). Selection for viable F1 hybrids is facilitated using complementary auxotrophic or antibiotic selection (for example, *ura3 MATa* mated to *lys2 MATɑ*; *ura3*/ + + /*lys2 MATa*/*MATɑ* selected on C-URA-LYS). Once successful hybrids are obtained, two other issues may arise. First, in the initial generations after hybridization, genomic instability may result in aneuploidy in the diploid hybrid. While the rate of aneuploidy in hybrid formation is unclear, anecdotal evidence suggests that it is quite common. Mutation accumulation experiments have demonstrated variable results, with some interspecific hybrids having increased point mutation rates, increased ploidy, and/or increased genomic instability (Dong et al. 2019; Fijarczyk et al. 2021; Tattini et al. 2019). If a euploid hybrid genome is important to your experimental design, sequencing independent hybrid matings is recommended.

Another factor to consider is mitochondrial inheritance. Mitochondria are typically uni-parentally inherited, but go through several generations of heteroplasmy where mitochondrial recombination may occur. Which parental mitotype becomes the homoplastic mtDNA depends on a spectrum of known and unknown genetic and environmental factors (Hewitt et al. 2020; Hsu and Chou 2017; Verspohl et al. 2018). In my own experience, otherwise isogenic *S. cerevisiae* × *S. uvarum* hybrids mated under the exact same environmental conditions yielded a mix of hybrids with either *S. uvarum* or *S. cerevisiae* mtDNA (Smukowski Heil et al. 2021). This is important, as mitotype plays a crucial role in a variety of phenotypes, including temperature tolerance and hybrid incompatibility (Baker et al. 2019; Jhuang et al. 2017; Lee et al. 2008; Li et al. 2019; Vijayraghavan et al. 2019). Species mtDNA can be assessed using primers (Baker et al. 2019) or whole genome sequencing. Note that mtDNA copy number and whole genome sequencing coverage is not accurately obtained with traditional DNA extraction methods, and a reference mtDNA genome does not exist for all *Saccharomyces* species. If one wants to control which species’ mtDNA is inherited, this is relatively easily done by creating ⍴^0^ mutants (lacking mtDNA) via passage on ethidium bromide (Fox et al. 1991).

**Fig. 1:**
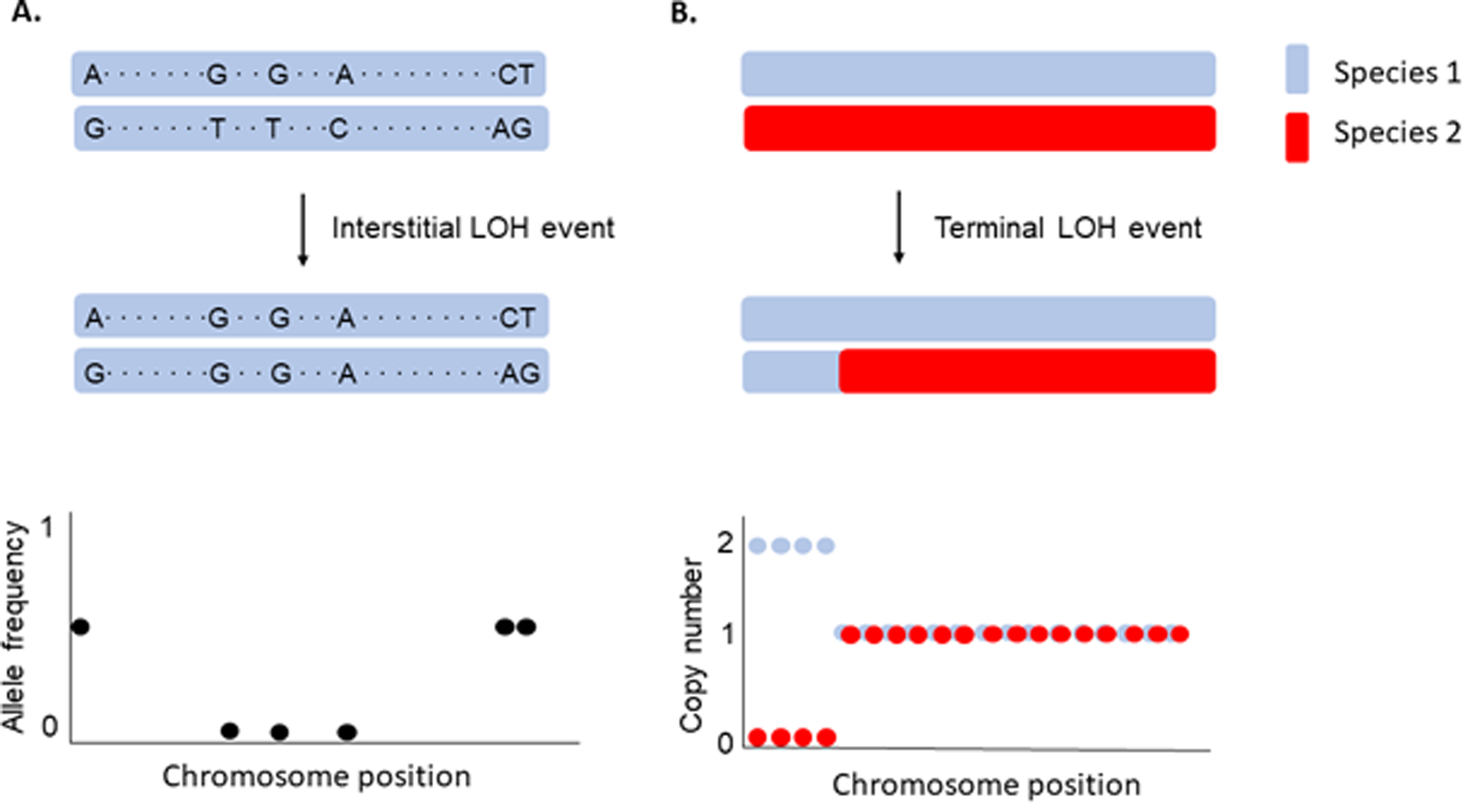
Detecting loss of heterozygosity in intraspecific diploids and interspecific diploids. **A**. An interstitial LOH event is depicted in an intraspecific hybrid diploid, detected by a change in allele frequency from 0.5 to 0 at several consecutive variants. **B**. A terminal LOH event is depicted in which an interspecific hybrid diploid (Species 1 in blue, Species 2 in red) has a LOH event that extends to the telomere. It is detected by a change in copy number from 1 copy of the locus in each species, to Species 1 having a copy number of 2 and Species 2 having a copy number of 0

The typical pipeline for detecting LOH in hybrids aligns sequencing reads to separate or concatenated parental reference genomes. This can be done in the same way as intraspecific hybrids, or using a pipeline designed for hybrid genome analysis like sppIDer (Langdon et al. 2018) or MuLoYDH (Tattini et al. 2019). Instead of using allele frequency shifts, copy number variants are used to identify hybrid LOH (Fig. 1B). A hybrid LOH event always appears as a copy number variant, either by one species’ allele replacing the other species (copy number amplified from one to two in one species and lost from one to zero in the other species in a diploid hybrid), or the LOH event precedes or happens in tandem with amplification of one allele (copy number greater than two). Most copy number variant programs can be co-opted for analyzing hybrid genomes, though in most instances the parental sub-genomes should be analyzed separately. Similarly to intraspecific hybrid LOH, programs Y_MAP_ and CONTROL-FREEC are common choices for copy number variant analysis (Abbey et al. 2014; Boeva et al. 2012), and sppIDer can also assess hybrid LOH (Langdon et al. 2018).

Detecting hybrid LOH in an evolved population is complicated by differentiating between copy number and frequency of the copy number variant in the population. Sequencing clones from the population can help clarify this, and tools developed for detecting subclonal frequencies in tumors like Control-FREEC can also be utilized (Boeva et al. 2012). Sequencing coverage for one species will be zero (in the case of a clone), or otherwise differing from the base coverage (in a population). Interspecific LOH is best visualized by identifying homologs and identifying copy number in homologous genes to account for translocations and other loss of synteny (Smukowski Heil et al. 2017, 2019).

## Conclusion

While sex is rare in *Saccharomyces* yeasts and other facultative sexual organisms in the wild, LOH provides a mechanism with some similar properties, like creating new haplotype combinations, accelerating adaptation, and potentially shedding deleterious alleles. Experimental evolution of heterozygous yeasts has clearly demonstrated that the LOH rate is quite high, can be adaptive, can accelerate adaptation, and can impact many phenotypes. Using heterozygous strain backgrounds in evolution experiments can provide increased genetic variation, clarify dominance of mutations, and identify genetic by environment interactions. Given the high levels of heterozygosity found in human-associated niches, identifying recurrent LOH in domesticated strains can even identify potential novel beneficial alleles for use in industry. With increasing interest in interspecific hybrids, and availability of more diverse strains than ever before, the future of experimental evolution using heterozygous strain backgrounds holds a lot of promise.
